# High Hydrostatic Pressure and Co-Fermentation by *Lactobacillus rhamnosus* and *Gluconacetobacter xylinus* Improve Flavor of Yacon-Litchi-Longan Juice

**DOI:** 10.3390/foods8080308

**Published:** 2019-08-01

**Authors:** Huali Chen, Gengsheng Xiao, Yujuan Xu, Yuanshan Yu, Jijun Wu, Bo Zou

**Affiliations:** Sericultural & Agri-Food Research Institute, Guangdong Academy of Agricultural Sciences, Key Laboratory of Functional Foods, Ministry of Agriculture and Rural Affairs, Guangdong Key Laboratory of Agricultural, Products Processing, Guangzhou, Guangdong Province 510610, China

**Keywords:** high hydrostatic pressure, *Lactobacillus rhamnosus*, *Gluconacetobacter xylinus*, free amino acids, volatile flavor compounds

## Abstract

The aim of this study was to evaluate the effect of high hydrostatic pressure (HHP) and co-fermentation by *Lactobacillus rhamnosus* and *Gluconacetobacter xylinus* on the quality of yacon-litchi-longan (YLL) juice. The carbohydrates, organic acids, free amino acids (FAAs), and volatile compounds in YLL juice were analyzed. Thermal processing (TP) increased the content of total carbohydrates, organic acids and FAAs, and destroyed the aroma components, whereas HHP treatment had a negligible effect. Carbohydrate content was lower, and content of lactic acid, acetic acid, and exopolysaccharide (EPS) were higher in co-fermented juice than in unfermented juice. Furthermore, the content of bitter FAAs in fermented TP and HHP-treated YLL juices decreased by 88.7% and 86.9%, respectively. Co-fermentation also increased ketones and the sum of individual volatile constituents, and improved the overall flavor of juice. Taken together, HHP treatment prior to co-fermentation can be used to improve the quality of YLL juice, especially the flavor thereof.

## 1. Introduction

Probiotics are an archetypal functional food and are beneficial to the host, improving the intestinal microbial balance. However, probiotic fermented dairy products can cause problems, such as lactose intolerance, an increase in cholesterol content, and a milk protein allergy. Hence, demand for probiotic products based on various plant-derived food substrates is increasing, especially for those based on fruits and vegetables [1,2].

Yacon (*Smallanthus sonchifolius* Poepp. Endl.) is an Andean root with known medicinal properties, which possesses a sweet taste and crisp texture and is consumed raw, cooked, baked or as juice. Yacon is rich in fructooligosaccharide (FOS), which improves the viability of probiotics in the digestive tract [3,4]. Litchi (Litchi chinensis Sonn.) and longan (Dimocarpus longan Lour.) are subtropical and tropical fruits. They are usually used to make natural juice drinks because of their attractive color, characteristic aroma, and sweet-sour mouth feel. Litchi and longan pulp are rich in polysaccharides and polyphenols, which exhibit many health benefits such as removal of free radicals. Combination of multiple fruits and vegetables for fermentation may enhance nutrient use rate of microbial populations, thus affecting their growth and metabolism [5].

Among the probiotics most studied in plant products, *Lactobacillus rhamnosus* (*L. rhamnosus*) stands out for viability and stability in fruit, fruit salad, and vegetable products. *Gluconacetobacter xylinus*, an Acetobacter, is a model microorganism for producing bacterial cellulose (BC) and acetic acid [6]. BC as a food ingredient has no adverse effect on the sensory characteristics of food, such as color or flavor. Currently, BC is used as a dietary source for desserts, fruit cocktails and jelly [7]. The above microbial species are considered nontoxic food-grade microorganisms. Co-fermentation of mixed microorganisms provides complex growth patterns and can considerably affect the organoleptic, nutritional and functional properties of food, for example, *L. rhamnosus* produced lactic acid, *Gluconacetobacter xylinus* (*G. xylinus*) produced acetic acid and BC. Our previous studies showed that *L. rhamnosus* grew well in yacon, litchi, and longan juice, and co-fermentation with *G. xylinus* increased its gastrointestinal tolerance. However, studies on the effect of co-fermentation on the physicochemical properties and flavor of multi-fruit juice are limited.

Fruit juices are traditionally pasteurized via thermal processing (TP) before microbial fermentation [8]. Previous studies reported that TP leads to large nutritional losses, poor color, and significant changes in the flavor of juices due to high temperature [9]. Generally, high hydrostatic pressure (HHP) sterilization is another option for juice processing. It is a novel non-thermal food processing method and known for inactivating food-borne spoilage and pathogenic microorganisms, without causing significant loss of food sensory and nutritional value [10,11,12]. However, the use of HHP-treated fruit juices as substrates for co-fermentation by mixed microorganisms has not been evaluated. 

Considering the above points, we hypothesized that HHP sterilization can maintain the quality of fresh juice, and co-fermentation may alleviate the adverse effects of heat treatment. The aim of the present study is to study the effects of HHP sterilization on the quality of fruit juice and that of the co-fermentation of *L. rhamnosus* and *G. xylinus* on the physicochemical properties of yacon-litchi-longan (YLL) juice. The results will provide a scientific basis for developing the YLL juice industry. 

## 2. Materials and Methods

### 2.1. Chemicals and Reagents

De Man Rogosa and Sharpe broth/agar (MRS), plate count agar, rose Bengal agar, and yeast extract were purchased from Guangdong Huankai Microbiology Technology Co., Ltd. (Guangzhou, China). Polypeptone, glucose fructose, sucrose, acetic acid, citric acid, lactic acid, malic acid, oxalic acid, and L-ascorbic acid were purchased from Shanghai Yuanye Biotechnology Co., Ltd. (Shanghai, China). 1-Kestose, nystose and 1F-fructofuranosylnystose were obtained from Wako Pure Chemical Industries, Ltd. (Tokyo, Japan). Mass spectrometry (MS)-grade acetonitrile, cyclohexanone, and methanol were obtained from Merck (Darmstadt, Germany). Pure water was obtained using a Milli-Q system (Bedford, MA, USA). Phenol and acetocaustin were obtained from Aladdin Reagent Co., Ltd. (Shanghai, China). Other reagents were of analytical grade and were purchased from Sinopharm Chemical Reagent Co., Ltd. (Shanghai, China). 

### 2.2. YLL Juice Preparation

YLL juice was made from three kinds of fruit: yacon, litchi and longan. Yacon juice was made from fresh yacon by peeling, slicing (5 mm thick), bleaching (at 100 °C, 2 min), soaking (in 0.40% vitamin C solution for 10 min), squeezing, and filtering. Litchi juice was purchased from the Fruit Fragrance Garden Food Co., Ltd. Longan juice was made from fresh longan by successive peeling and core-removing, squeezing, and filtering. Finally, yacon, litchi, and longan juices were mixed (1:1:1, *v*/*v*/*v*) and stored at −20 °C. 

### 2.3. Preparation of Cultures

The commercial probiotic cultures *L. rhamnosus* (Lr-G14) and the *G. xylinus* ATCC23767 were purchased from Guangzhou Shuowei Food Technology Co., Ltd. and Guangdong Microbial Culture Center (GDMCC), respectively. The commercial cultures were activated according to the manufacturer’s instructions. Stock culture of *L. rhamnosus* was prepared by mixing activated MRS broth and 40% glycerol (*v*/*v*) in 1:1 ratio in 1 mL in cryovials and freezing at −20 °C. For *G. xylinus*, acetobacter medium #350 (5 g/L yeast extract, 5 g/L polypeptone, 5 g/L glucose, 5 g/L mannitol, 5 mL/L ethanol absolute, and 1 g/L MgSO4·7H2O pH 6.6-7.0) was mixed with 40% glycerol (*v*/*v*) in 1:1 ratio in 1 mL cryovials and frozen at −20 °C. The lactobacillus (LAB) strains and *G. xylinus* were reactivated by inoculating them onto MRS broth and acetobacter medium #350, respectively, and incubating for 18 h at 30 °C for LAB and for 24 h at 30 °C and 160 r/min for *G. xylinus*. 

### 2.4. Processing Conditions

To compare TP and HHP, the processing conditions were selected for equivalent microbial inactivation (commercial aseptic standard). TP was conducted at 100 °C for 30 s, and the HHP treatment was performed in a bench-scale high-pressure homogenizer (RLGY-600; Wenzhou Nobel Machinery Co., Ltd., Wenzhou, China). YLL juice was packaged and vacuum-sealed in aluminum foil bags. Samples were subjected to pressures of 300, 400, or 500 MPa for 15 min at 25 °C. The TP-treated YLL juice and HHP-treated YLL juice were then stored at −20 °C until further experiment and analysis. 

### 2.5. Fermentation Assays

The TP- and HHP-treated YLL juice were thawed and then inoculated with 6 log colony-forming units (CFU)/mL *L. rhamnosus* and 10% (*v*/*v*) *G. xylinus*. The fermentations were performed in 500 mL Erlenmeyer flasks containing 400 mL of the substrates at a rotational speed of 160 r/min at 30 °C for 48 h. The experiments contained five groups: fresh YLL juice (Fresh), TP-treated YLL juice (TP), fermented TP-treated YLL juice (F-TP), HHP-treated YLL juice (HHP) and fermented HHP-treated YLL juice (F-HHP). Samples (20 mL) were taken at 0, 12, 24, 36, and 48 h of fermentation for subsequent analysis. The experiments were performed in triplicate and two samples were taken for each repetition.

### 2.6. Microbiological Assay

The number of residual viable microorganisms in HHP-treated YLL juice sample was measured using an official Chinese method [13]. Samples (1 mL) were diluted serially ten-fold in a solution of 0.9% NaCl (*w*/*v*). Each sample was spread onto plate count agar medium and rose Bengal agar to determine the viable aerobic yeast, and mold plate counts, respectively. The incubation conditions for aerobic bacteria were 1–2 days at 37 °C, and the incubation conditions for yeast and mold were 4–5 days at 28 °C. The viable count of LAB in fermented YLL juice was determined per an official Chinese method [14]. Samples (1 mL) from each fermentation flask were serially diluted ten-fold in a solution of 0.9% NaCl (*w*/*v*). The total LAB population was determined by plating in MRS agar. Plates were incubated for 1–2 days at 37 °C. The colony-forming units (CFU) were enumerated in plates containing 30 to 300 colonies, and the result was expressed as log CFU/mL. The analyses were carried out in triplicate. 

### 2.7. Chemical Analysis

#### 2.7.1. Carbohydrate

Carbohydrates were analyzed using an High performance liquid chromatography (LC-20AT; Shimadzu Corp., Tokyo, Japan) equipped with a low temperature evaporative light scattering detector (ELSD-LT2; Shimadzu). Carbohydrates were separated on a Shodex packed column (Asahipak NH2P-50 4E, 4.60 mm × 250 mm) using 70% acetonitrile [15]. The column temperature was 40 °C and the drift tube temperature was 50 °C. The injection volume was 10 μL and the flow rate was 1.0 mL/min. The compounds were identified based on the retention time of standards, and their concentrations were calculated from the external calibration curves of standards. All samples were examined in triplicate. 

#### 2.7.2. Organic Acids

Organic acids were analyzed using an HPLC with photodiode array detection (LC-20AT; Shimadzu Corp., Tokyo, Japan). An Agilent packed column (ZORBAX SB-C18, 4.60 mm × 250 mm) was used for organic acid separation (30 °C) [15](Yu et al., 2015). The column temperature was 30 °C and the mobile phase was 0.10 mol/L (NH4)2HPO4 (pH 2.70). The flow rate was 0.6 mL/min and the injection volume was 10 μL. Ascorbic acid was detected at 245 nm and other organic acids were detected at 210 nm. The content of organic acids was calculated from the external calibration curves of standards. All samples were examined in duplicate. 

#### 2.7.3. Exopolysaccharide (EPS)

EPS was extracted and determined according to the previous report [16]. Briefly, the samples (600 μL) were mixed with 300 μL of 40% (*w*/*v*) trichloroacetic acid aqueous solution, stored at 20 °C for 10 min and then transfer to 4 °C for 2 h. After centrifugation (12,000 *g* for 5 min at 4 °C), EPS was precipitated by chilled ethanol (100%, *v*/*v*, −20 °C) and maintained at 4 °C for 14 h. The precipitate was collected and dissolved in 1 mL deionized water (60 °C). EPS concentration was measured using the phenol-sulfuric acid method.

#### 2.7.4. Free Amino Acids (FAA)

As described by Ibegbulem [17]), 1 mL sample was added to 1 mL 5-sulfosalicylic acid solution (6%, *m*/*v*) for 2 h at 4 °C to remove large peptides. Then, 0.5 mL 0.06 mol/L HCl solution and 0.5 mL 1% EDTA-2Na were added. The sample was thoroughly mixed and centrifuged for 10 min at 10,000 *g* (4 °C). One milliliter supernatant was added to 2 mL sodium citrate buffer, pH 2.2, mixed evenly and centrifuged. Supernatant (20 μL) was injected into an L-8900 automatic amino acid analyzer (Hitachi, Tokyo, Japan). The FAA content in juice sample was calculated by calibrating with standard amino acids using the EZChrom Elite System. 

#### 2.7.5. Volatile Flavor Compounds

Each juice sample (6 g) was added to 15 mL headspace glass vials with 3 g sodium chloride, 20 μL internal standard cyclohexanone (10 μg/mL in absolute methanol) [12], a nd a magnetic stirring rotor, and incubated for 20 min at 40 °C and 15 r/min. Volatile compounds were extracted and adsorbed at 40 °C for 40 min using a Divinylbenzene/Carboxen/Polydimethylsiloxane (DVB/CAR/PDMS) fiber (50 μm), and then desorbed at 270 °C into the gas chromatograph inlet with a sampler for 5-6 min. A gas chromatography mass spectrometer (GC-MS) system (6890N /5975B; Agilent Technologies Co., Ltd.; Santa Clara, CA, USA) equipped with a DB-5MS elastic capillary column (30 m × 0.25 mm × 0.25 um; Agilent Technologies, Santa Clara, CA, USA), and non-diversion injection mode was used. Helium carrier gas was delivered at a flow rate of 1.7 mL/min. The mass detector was operated in electron impact mode (70 eV) and the temperature of the ion source was 230 °C. The extracted volatile compounds were identified by comparing the mass spectra with those in mass spectral libraries (NIST16). The retention indexes (RI) of unknown compounds were determined using sample injection of n-alkanes (C8-C20). The quantity of volatile compounds was determined using the internal standards. Tentative identification was performed by comparing mass spectra and RI from literature. Volatile compounds were partially quantitated using Equation (1):

(1) $$C(\mu g/g)=\frac{A_{C}}{A_{\mathrm{is}}}C_{\mathrm{is}}\left(\mu g/g\right)$$

where C is the relative concentration of the analyzed sample, C_is_ is the final concentration of the internal standard in sample, A_c_ is the peak area of the analyzed sample, and A_is_ is the peak area of the internal standard.

### 2.8. Statistical Analysis

The results were expressed as mean value ± standard deviation (SD). Data were analyzed using one-way analysis of variance (ANOVA) of SPSS 17.0 (Chicago, IL, USA), followed by Tukey’s multiple-range test. The mean difference was considered significant at *p* < 0.05. Volatile compounds were analyzed using principal component analysis (PCA) and partial least squares discrimination analysis (PLS-DA) of SMICA Ver. 14.1 (Umetrics Inc., Malmö, Sweden). Figures were prepared using Origin Ver. 8.5 (Origin Lab Inc., Northampton, MA, USA). 

## 3. Results and Discussion

### 3.1. Effect of HHP Treatment on Inactivation of Total Aerobic Bacteria, Yeast, and Mold in YLL Juice

**Figure 1:**
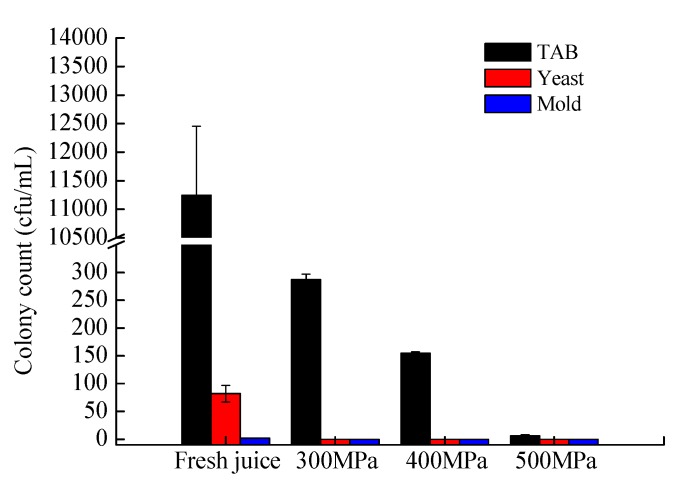
Changes in total aerobic bacteria (TAB), yeast and mold counts of fresh yacon-litchi-longan (YLL) juice as treated by high hydrostatic pressure (HHP) (300 MPa, 15 min), HHP (400 MPa, 15 min) and HHP (500 MPa, 15 min).

The three juices ran a high risk of microbial contamination as the residual indigenous microorganisms in their hull can easily pass into the juice during juice preparation. The number of indigenous microorganisms in YLL juice was more than 4.0 log CFU/mL, and the number of yeasts reached 2.0 log CFU/mL (Figure 1). Therefore, fresh YLL juice should undergo sterilization to ensure product quality and safety prior to fermentation. Previous studies have shown that the total number of viable microorganisms in fresh YLL juice after TP (TP; 100 °C, 30 s) met the commercial aseptic standard. After individually treating with HHP at 300 MPa for 15 min, 400 MPa for 15 min, and 500 MPa for 15 min, yeast and mold were completely inactivated, and total aerobic bacterial counts decreased to 2.5, 2.2, and 0.8 log CFU/mL (Figure 1), respectively. Results indicated that YLL juice treated with HHP (500 MPa, 15 min) met the commercial aseptic standard. 

### 3.2. Microbial Growth and Substrate Consumption of TP- and HHP-Treated YLL Juice During Co-Fermentation

As shown in Figure S1, *L. rhamnosus* grew rapidly in the juice, and *L. rhamnosus* counts reached a maximum of 9.48 log CFU/mL in F-HHP juice and 9.49 log CFU/mL in F-TP juice at the end of fermentation (Figure S1). Producing juices of quality closely similar to that of fresh-squeezed juices and ensuring constant original juice quality are the main challenges in the juice industry. As shown in Table 1, the carbohydrate content in YLL juice increased significantly after TP treatment (*p* < 0.05), whereas it did not change after HHP treatment (Table 1). This is probably a consequence of the loss of water in the juice caused by heating. HHP-treated YLL juice maintained almost the same component content as fresh YLL juice. After co-fermentation with *L. rhamnosus* and *G. xylinus* for 48 h, the glucose, fructose, and sucrose content of F-HHP juice was partially reduced, whereas the FOS (1-kestose, nystose, and 1F-fructofuranosylnystose) content changed slightly (Table 1). Some studies have shown that with the exception of Lactobacillus acidophilus, Lactobacillus casei, and Lactobacillus plantarum, not all Lactobacillus can use FOS. *L. rhamnosus* as a widely studied probiotic strain cannot use FOS [18]. In addition, EPS content increased to 67.02 mg/L (Table 1). Similar results were also observed in F-TP juice after 48 h of co-fermentation with *L. rhamnosus* and *G. xylinus*. The EPS content eventually reached 56.71 mg/L, which was significantly lesser than that of F-HHP juice (*p* < 0.05) (Table 1). After 48 h of co-fermentation, the use of carbohydrate in F-TP juice was 1.38% higher than in F-HHP juice.

In addition, HPLC analysis of organic acids showed that lactic acid and acetic acid were the main metabolites produced by *L. rhamnosus* and *G. xylinus*, respectively. After 48 h of co-fermentation, lactic acid concentration in F-TP and F-HHP juice reached 4.26 and 2.89 g/L, respectively, whereas acetic acid concentration reached 1.0 and 0.77 g/L, respectively (Table 1). The citric acid in F-TP and F-HHP juice was almost completely consumed after 48 h of co-fermentation. A significant decrease in malic, oxalic, and ascorbic acid contents were also observed (Table 1). It reported that *L. rhamnosus* converts malic acid into lactic acid via malic acid-lactic acid fermentation (malolactic fermentation, MLF) [19]. The increase of acetic acid is due to *G. xylinus* activity. 

### 3.3. Changes in FAA Content of TP- and HHP-Treated YLL Juice After Co-Fermentation

**Figure 2:**
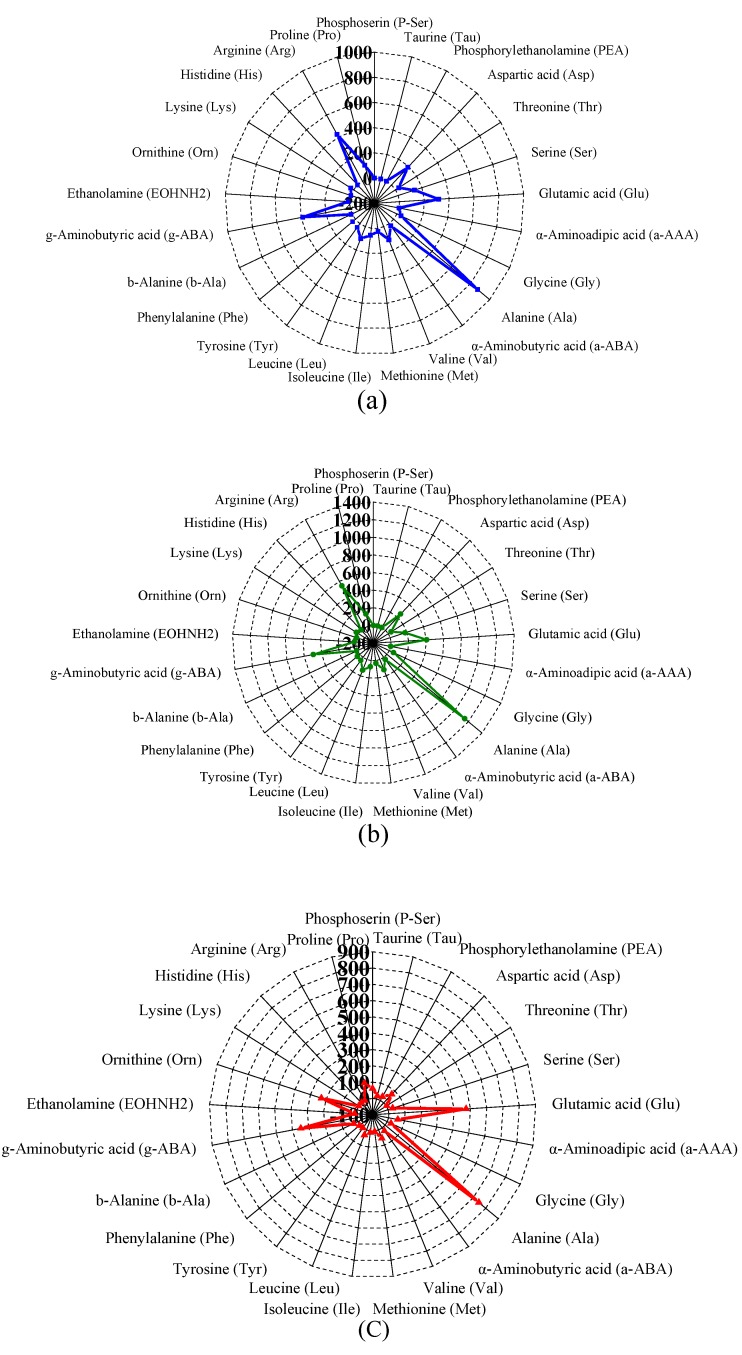

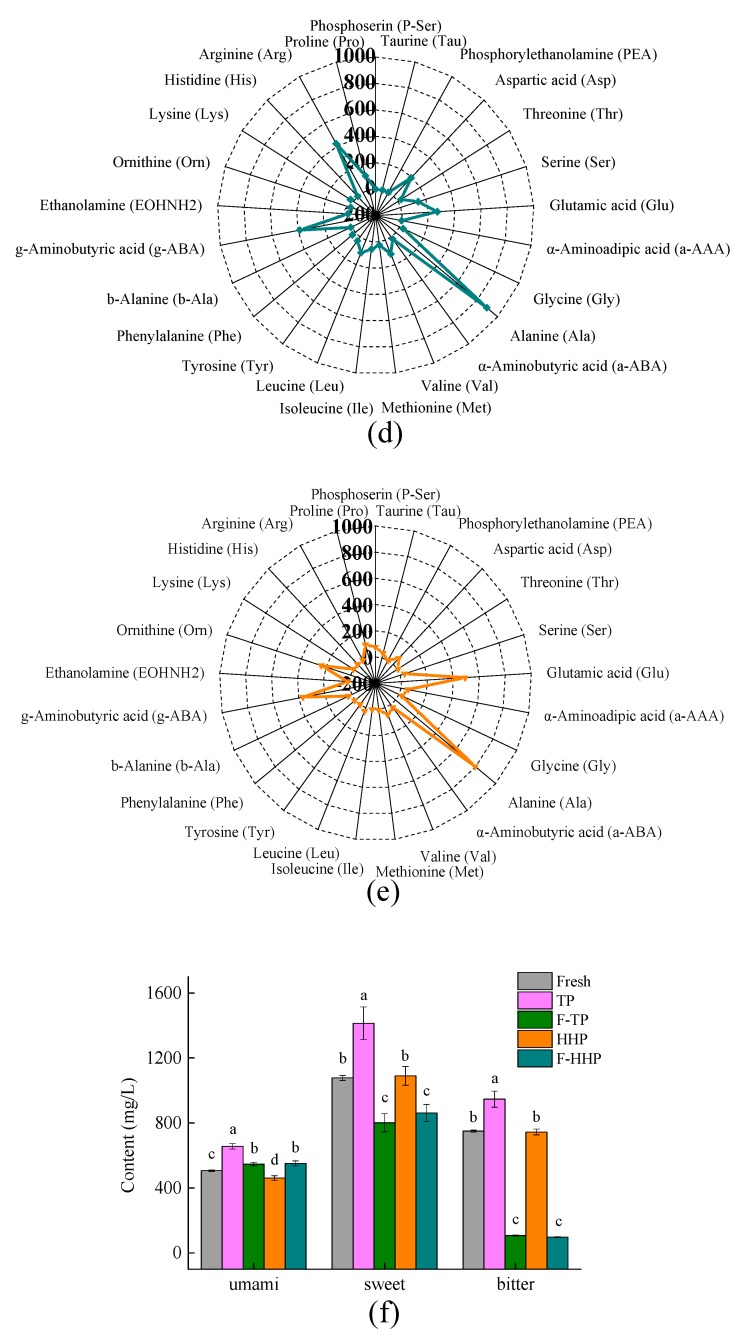
Free amino acid (**FAA**) in different treated yacon-litchi-longan (YLL) juice. FAA content (mg/L) in the (**a**) fresh juice, (**b**) thermal processing treated YLL juice (TP), (**c**) fermented thermal processing treated YLL juice (F-TP), (**d**) high hydrostatic pressure treated YLL juice (HHP), and (**e**) fermented high hydrostatic pressure treated YLL juice (F-HHP), (**f**) Total content of FAA with taste characteristics in different treated YLL juice (Different letters represented a significant difference within the same taste characteristic (*p* < 0.05)).

Previous studies reported that different food processing technologies can alter the composition of amino acids, for example, heating can modify the amino acids themselves [20]. This study analyzed the changes in FAAs in YLL juice treated using different sterilization processes and co-fermentation. As shown in Table S1, YLL juice includes almost all the essential amino acids and non-essential amino acids, although their content varied in the five groups. The total content of FAAs in fresh, TP, and HHP juice were approximately 2894.62, 3777.22, and 2892.39 mg/L, respectively (Table S1). A significant increase in total FAA content was observed in TP juice (*p* < 0.05), which is possibly caused by the evaporation of water during heating, whereas no significant difference in the content of FAAs was observed between HHP juice and fresh juice (*p* > 0.05) (Table S1). Blandino et al. [21] reported that the results of many studies on the effects of fermentation on protein and amino acid levels are controversial. After co-fermentation using *L. rhamnosus* and *G. xylinus*, the total content of FAAs in F-TP and F-HHP juice were approximately 2353.07 and 2494.58 mg/L, respectively (Table S1), which corresponded to 30.40% and 13.75% decrease. Some FAAs (Aspartic acid (Asp), Threonine (Thr), Serine (Ser), Valine (Val), Methionine (Met), Isoleucine (Ile), Leucine (Leu), Tyrosine (Tyr), Phenylalanine (Phe), Lysine (Lys), and Arginine (Arg)) were used significantly (*p* < 0.05), whereas the content of certain FAAs (Phosphoserine (P-ser), Taurine (Tau), α-aminoadipic acid (a-AAA), β-Alanine (b-Ala) and Ornithine (Orn)) increased significantly (*p* < 0.05) in the F-TP and F-HHP juice. Slight changes in other FAA concentrations were also observed. The highly abundant FAAs in fresh, TP, and HHP juice were Asp, Alanine (Ala), g-aminobutyric acid (g-ABA), Glutamic acid (Glu) and Arg (Figure 2a,b,d). The highly abundant FAAs in F-TP and F-HHP juice were Glu, Ala, g-ABA, and Orn (Figure 2c,e). The taste characteristics of FAA can be categorized into umami (containing Asp and Glu), sweet (containing Gly, Ser, Ala, and Thr), and bitter (containing Arg, Ile, Leu, Met, Histidine (His), Phe, and Val) [22]. Compared to fresh YLL juice, TP- and HHP-treated samples, as well as fermented juices, exhibited similar levels of umami FAA. The contents of sweet and bitter FAA were significantly higher in TP juice than in fresh YLL juice (*p* < 0.05), whereas no significant difference was observed between HHP and fresh juice (*p* > 0.05) (Figure 2f). Compared to the fresh YLL juice, TP destroyed the natural taste of juice by changing the taste components. However, HHP treatment had negligible effect on the flavor of YLL juice. After co-fermentation, F-TP and F-HHP juices exhibited a 43% and 21% decrease, respectively, in the total content of sweet FAAs, and an 89% and 87% decrease, respectively, in the total content of bitter FAAs (*p* < 0.05) (Figure 2f). These results indicated that co-fermentation can improve the taste of YLL juice by changing amino acid composition.

### 3.4. Effect of HHP and Co-Fermentation on Volatile Flavor Compounds in YLL Juice

**Figure 3:**
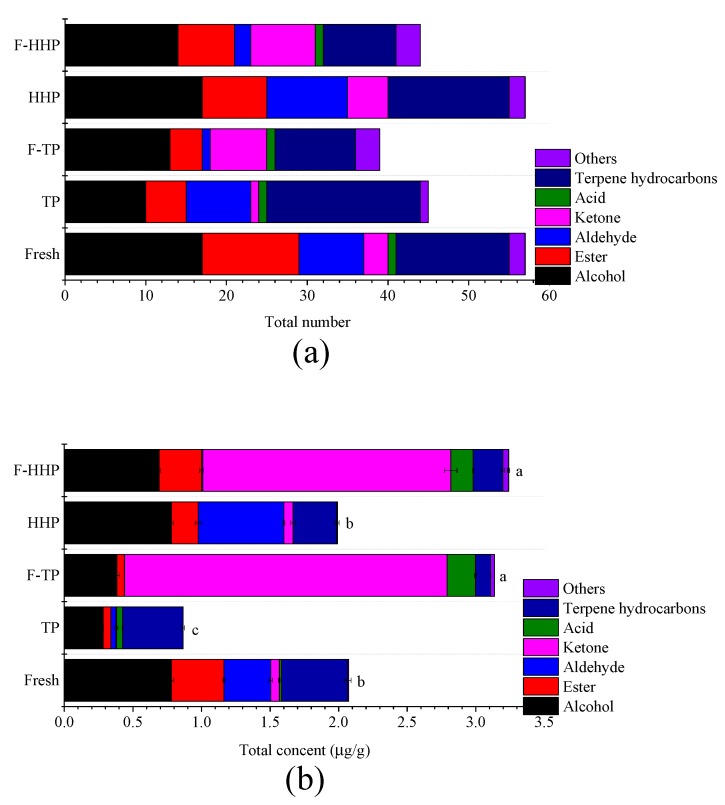
(**a**) Total number and (**b**) content of different volatile flavor compounds in different treated yacon-litchi-longan (YLL) juice. Different letters represented a significant difference within the same row (*p* < 0.05).

The volatile compounds of all treated YLL juices were determined using headspace solid phase microextraction gas chromatography mass spectrometer (HS-SPME-GC-MS), which simultaneously integrates the extraction, concentration, isolation, and identification of volatiles via a solvent-free technique with time- and labor-saving characteristics. In total, 95 volatile compounds were identified and quantified in the headspace phase of all treated groups, including alcohols, esters, aldehydes, ketones, acids, phenols, terpene hydrocarbons, and others (Table S2). The content of each compound was calculated on the basis of the peak areas normalized using Equation (1). The volatile compounds strongly influenced the taste and odor of YLL juice, and sterilization and fermentation considerably altered the flavor profile of the final juice. HHP processing could change the formation of flavor compounds because chemical and enzymatic reactions maybe induced by high pressure [23]. As shown in Table S2, 57 volatile compounds were detected both in fresh juice and HHP juice, including 17 alcohols, 12 esters, eight aldehydes, three ketones, one acid, 14 terpene hydrocarbons, and two others in fresh juice, and 17 alcohols, eight esters, 10 aldehydes, five ketones, 15 terpene hydrocarbons, and two others in the HHP juice. The number of esters in YLL juice decreased after HHP treatment, which may be attributed to activation or inactivation of enzymes related to ester synthesis, induction of esters hydrolysis, and change in chemical composition and microstructure [12,24]. Forty-one compounds were detected in TP juice, including 10 alcohols, five esters, eight aldehydes, one ketone, one acid, 19 terpene hydrocarbons, and one other compound. Compared to HHP treatment, TP treatment considerably reduced the number of flavor substances in YLL juice, especially alcohols and esters (Figure 3a). Alcohols contribute significantly to the overall aroma in the form of solvents for other aromatic compounds, whereas esters contribute to fruity odor in fruit [25,26]. Therefore, the decrease in the number of volatile compounds confirmed that TP negatively affected the perception of juice flavor. The results are in line with most existing literature. Perez-Cacho and Rouseff [27] reported that aroma of orange juice was affected by the loss of compounds caused by a complex series of chemical reactions after heat treatment. Altogether, TP treatment, but not HHP treatment, changed a large number of distinct volatile compounds in YLL juice. After co-fermentation by *L. rhamnosus* and *G. xylinus*, the total number of volatile substances, represented by aldehydes, esters, and terpene hydrocarbons decreased, whereas those of ketones increased (Figure 3a).

The total content of volatile compounds in fresh YLL juice was 2.072 μg/g, which was significantly higher than that in TP-treated YLL juice (*p* < 0.05). Compared to fresh juice, the highest loss of volatile flavor compounds occurred in TP (0.875 μg/g), followed by HHP juice (1.996 μg/g), corresponding to 57.77%, and 3.67% decrease, respectively (Figure 3b). However, the increase in the content of volatile flavor compounds, attributable to the increase in ketone volatile compound content, was observed in F-TP (3.137 μg/g) and F-HHP juices (3.246 μg/g), corresponding to 51.40% and 56.47% increase, respectively, indicating that fermentation significantly increased the flavor component content in YLL juice (*p* < 0.05) (Figure 3b). Ketones accounted for 75.01% and 55.82% of the total volatile fraction in F-TP and F-HHP juices, respectively, and were the major classes of volatiles (Figure 3b). This result is consistent with the observations of many previous studies [25], which showed high levels of ketones, especially 2, 3-butanedione (diacetyl) and 3-hydroxy-2-butanone (acetoin) in several fermented fruit and vegetable juices. Diacetyl and acetoin can be bio-synthesized starting from citrate existing in many substrates such as fruit [28,29]. Ketones tend to provide fruity, herbaceous, and banana-like aromas [30]. Compared to non-fermented samples, co-fermentation using *L. rhamnosus* and *G. xylinus* produced some new ketones, especially high amounts of 2, 3-butanedione and 3-hydroxy-2-butanone. These ketones are related to pleasant flavor characteristics; for example, 2, 3-butanedione has fruity flavor, 3-hydroxy-2-butanone has milky aroma, 2-heptanone has pear-like odor, and 2-nonanone possesses the aroma of fruit, flowers, oils, and herbs. The content of aldehydes and terpene hydrocarbons decreased simultaneously in fermented YLL juice; in particular, aldehyde content decreased to nearly zero (Figure 3b). High concentrations of aldehydes may cause off-flavors as aldehydes are unstable compounds in food matrices that are easily reduced to alcohols or oxidized to acids by microorganisms [31,32]. The alcohol content in YLL juice changed slightly after co-fermentation, but the contents of linalool and geraniol increased a lot, and accounted for a higher proportion of alcohols (not show), thereby contributing flowery (rose-like) and fruity (citrus-like) notes. An increase in ester content was observed in F-HHP juice, which was mainly attributed to the high content of a newly synthesized ester, namely, ethyl caproate with koji and pineapple aroma. Ethyl caproate was also detected in F-TP juice. The increase in the level of esters in fermented juice may be related to the increased availability of alcohol precursors [33]. The increase in the content of acid volatile flavor compounds is attributed to the acetic acid produced by *G. xylinus* fermentation.

**Figure 4:**
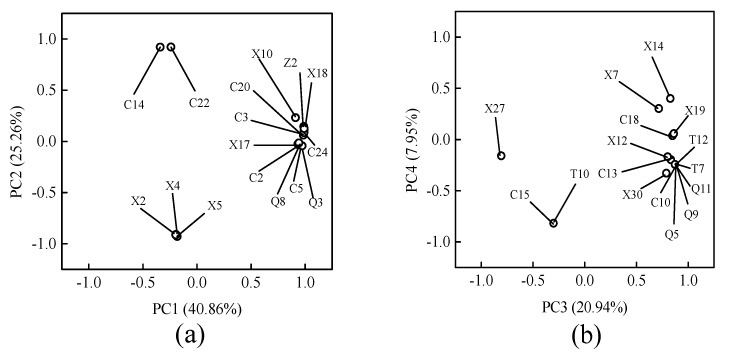
Diagram of principal component (PC) factor of volatile flavor compounds in different treated yacon-litchi-longan (YLL) juice (**a**) and (**b**). C2 = 3-Methyl-1-butanol; C3 = 2-Methylbutan-1-ol; C5 = trans-2-Hexen-1-ol; C10 = 1-Octanol; C13 = Bicyclo[3.1.1]hept-3-en-2-ol,4,6,6-trimethyl-; C14 = Neroloxide; C15 = Borneol; C18 = 2-(4-Methylphenyl)propan-2-ol; C20 = 2,6-Dimethyl-3,5,7-octatriene-2-ol, E,E-; C22 = 6-Octen-1-ol,3,7-dimethyl-, (3R)-; C24 = Geraniol; Z2 = 2-Butenoic acid, ethyl ester; Q3 = trans-2-Hexenal; Q5 = Heptaldehyde; Q8 = Octanal; Q9 = (E)-2-Octenal; Q11 = ampholenic aldehyde; T7 = (R,S)-5-Ethyl-6-methyl-3E-hepten-2-one; T10 = 2-Undecanone; T12 = 4-[2,2,6-trimethyl-7-oxabicyclo[4.1.0]hept-1-yl]-3-Buten-2-one; X2 = 3-Methyl-hexane; X4 = Heptane; X5 = Methylcyclohexane; X7 = Ethylbenzene; X10 = (1S)-(-)-alpha-Pinene; X12 = Decane; X14 = 1-methyl-3-(1-methylethyl)-benzen; X17 = 1-methyl-4-(1-methylethenyl)-Benzene; X18 = (3E,5E)-2,6-Dimethyl-1,3,5,7-octatetrene; X19 = 3,6-Dimethyl-2,3,3a,4,5,7a-hexahydrobenzofuran; X27 = d-Cadinene; X30 = 2,4-Di-tert-butylphenol.

**Figure 5:**
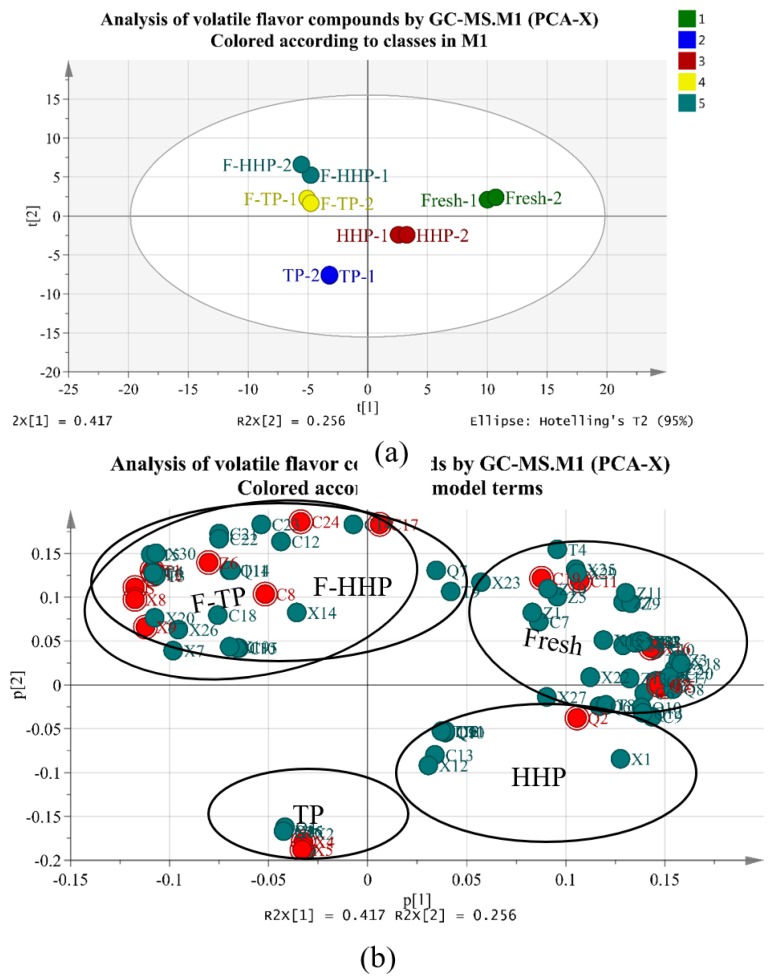
(**a**) principal component analysis (PCA) score plot and (**b**) loading plot of volatile flavor components of different treated yacon-litchi-longan (YLL) juice.

To correlate the volatile compound content with the different treated YLL juice, PCA and PLS-DA were performed, and contents of the 95 volatile flavor compounds in all YLL juice samples were taken as analytical variables. PCA reduced the dimensionality within the data set and detected similarities and/or differences among juice samples [34]. Table S3 shows that PCA and PLS-DA models have four principal components and the fitting parameters are R^2^X = 0.966, Q^2^ = 0.859; the PLS-DA model contains four principal components and the fitting parameters are R^2^X = 0.997, Q^2^ = 0.987. R^2^X is closer to 1, indicating that the model is stable, and Q^2^ > 0.8 indicates that the prediction rate is higher, which shows that the above two models completely reflect the overall information of the YLL juice of each treatment group. PCA results are shown in Figure 4 and Figure 5, which reveal the association between flavor compounds and different samples. Figure 4 shows a diagram of the PC (principal component) factor, where the first, second, third, and fourth PC explain 40.86%, 25.26%, 20.94%, and 7.95% of the total variance, respectively. The cumulative variance contribution rate was 95% and the information of all variables was almost included. Statistical analysis indicated that the correlation of volatile components in different treated YLL juice was high. Eleven compounds positively loaded on PC1, which was highly influenced by 2-methylbutan-1-ol (special odor), 2,6-dimethyl-3,5,7-octatriene-2-ol, E,E-, geraniol(flower aroma), 2-butenoic acid, ethyl ester (strong acid and fruit aromas with rum and ether aromas), and (3E,5E)-2,6-dimethyl-1,3,5,7-octatetrene. The positive axis of PC2 was closely related to neroloxide (roses and oranges aroma) and 6-octen-1-ol,3,7-dimethyl-, (3R)- (flower aroma). The negative axis of PC2 was closely related to 3-methyl-hexane, heptane, and methylcyclohexane. Thirteen compounds positively loaded on PC3, which was highly influenced by 1-octanol (lemon odor), heptaldehyde (fruit aroma), (E)-2-octenal (grass aroma), ampholenic aldehyde(cool, green, pine and camphor aroma), (R,S)-5-ethyl-6-methyl-3e-hepten-2-one (special aroma), and 4-[2,2,6-trimethyl-7-oxabicyclo[4.1.0]hept-1-yl]-3-buten-2-one (sweet and floral aroma). The negative axis of PC3 was closely related to d-cadinene. The negative axis of PC4 was closely related to borneol (camphor-like odor) and 2-undecanone (rutin-like and peach-like aroma). Figure 5 shows the score plot and loading plot. The distribution of scores in the five samples (fresh, TP, F-TP, HHP, and F-HHP) revealed five separate groups in YLL juice, reflecting the distinctive sensorial and flavor differences among the samples (Figure 5a). The loading plot (Figure 5b) shows the distribution of volatile components, which corresponds to the distribution of sample points in the score plot. In the loading plot, compound locations are far from the origin, indicating that they are important for the differentiation pattern for the observed sample distribution [35]. In particular, the F-HHP groups are less separated from the TP groups, indicating a possible similarity of volatile characteristics of these two classes (Figure 5a). To select discriminant volatile compounds, the variables whose absolute variable importance factor (VIP) values exceed threshold value of 1 are regarded as important variables and selected as discriminant variables. A VIP value can be correlated with a higher concentration of the selected discriminant compound in a certain treatment class compared to others. The VIP value of PLS-DA in the loading plot can quantify the contribution of each volatile component to the classification. The more the VIP value (VIP value > 1), the higher the contribution of the volatile components to the difference of YLL juice. According to the VIP values (red marker) of the PCA score plot and loading plot (Figure 5), the characteristic volatile components of the five groups of YLL juice are mainly as follows (Table S4): trans-2-hexenal, trans-2-hexen-1-ol, linalool, α-terpineol, (1S)-(-)-alpha-pinene, and (Z)-13,7-dimethyl-3,6-octatriene in fresh juice; cyclopentane,1,2-dimethyl-, heptane, and methylcyclohexane in TP juice; 2,3-butanedione, 3-hydroxy-2-butanone and acetic acid in F-TP juice; hexanal, heptaldehyde, and 1-octanol in HHP juice; 6-hepten-1-ol, 2-methyl-, terpinen-4-ol, geraniol, ethyl acetate, ethyl caproate, p-xylene, and phenylethylene in F-HHP juice. The characteristic volatile components of fresh YLL juice are mainly green grass, flower, fruity, and resin fragrance. The TP juice mainly possesses fatty and soapy-liked odor. The HHP-treated YLL juice mainly possesses green grass, lemon, and fruit fragrance. The F-TP juice mainly possesses butter and milk fragrance. The F-HHP juice has mainly wine, flower, fruit, and sweet fragrance. Aroma was not just a result of the contents of individual compounds, but the result of the odor threshold. Further studies are required to investigate the odor activity values using GC-MS and GC-olfactometry (GC-O).

## 4. Conclusions

This study investigated the effects of TP, HHP, and co-fermentation treatments on carbohydrate, organic acid, and FAA content, and flavor characteristics of YLL juice. Results indicated that YLL juice can be subjected to HHP treatment to ensure microbial safety. The nutritional quality and aroma characteristics of the HHP-treated juice were similar to that of fresh juice. After co-fermentation with *L. rhamnosus* and *G. xylinus*, lactic acid, acetic acid, and EPS contents increased, and those of bitter FAAs decreased. Furthermore, increase in aroma compound content, resulting in better flavor and aroma, and more FAA retention and EPS production, were observed in co-fermented HHP-treated YLL juice compared to co-fermented TP-treated YLL juice. In conclusion, our results indicated that HHP treatment does not affect the quality and flavor of YLL juice, and co-fermentation using *L. rhamnosus* and *G. xylinus* can be used as a valuable strategy for improving the quality of YLL juice, thereby enriching or replacing original fruit juice aroma, which is often compromised by processing.

## Figures and Tables

**Table 1:** Carbohydrate, exopolysaccharide (EPS), and organic acid content in different treated yacon-litchi-longan (YLL) juice.

	Fresh	TP	F-TP	HHP	F-HHP
Carbohydrates (g/L)	97.00 ± 0.06 ^d^	117.89 ± 0.15 ^a^	101.61 ± 0.11 ^b^	97.80 ± 0.06^c^	85.64 ± 0.22 ^e^
Glucose	20.78 ± 0.03 ^b^	25.14 ± 0.13 ^a^	16.86 ± 0.12 ^c^	21.11 ± 0.16 ^b^	15.71 ± 0.18 ^d^
Fructose	23.99 ± 0.06 ^c^	29.33 ± 0.14 ^a^	24.31 ± 0.03 ^b^	24.36 ± 0.03 ^b^	22.24 ± 0.02 ^d^
Sucrose	42.89 ± 0.00 ^c^	52.59 ± 0.26 ^a^	49.96 ± 0.28 ^b^	42.92 ± 0.06^c^	38.54 ± 0.25 ^d^
1-Kestose	3.97 ± 0.09 ^c^	4.72 ± 0.06 ^a^	4.46 ± 0.02 ^b^	3.88 ± 0.02 ^c^	3.60 ± 0.01 ^d^
Nystose	3.14 ± 0.01 ^cd^	3.73 ± 0.03 ^a^	3.63 ± 0.01 ^b^	3.21 ± 0.03 ^c^	3.13 ± 0.03 ^d^
1^F^-Fructofuranosylnystose	2.24 ± 0.02 ^c^	2.43 ± 0.02 ^a^	2.41 ± 0.09 ^b^	2.24 ± 0.00 ^c^	2.19 ± 0.01 ^c^
EPS (mg/L)	34.81 ± 074 ^d^	48.25 ± 7.59 ^c^	56.71 ± 3.43 ^b^	34..46 ± 0.92 ^d^	67.02 ± 4.14 ^a^
Organic acids (g/L)					
Oxalic acid	0.30 ± 0.00 ^c^	0.35 ± 0.00 ^a^	0.16 ± 0.00 ^e^	0.34 ± 0.00 ^b^	0.17 ± 0.00 ^d^
Acetic acid	0.31 ± 0.01 ^c^	0.38 ± 0.01 ^c^	1.00 ± 0.05 ^a^	0.32 ± 0.01 ^c^	0.77 ± 0.07 ^b^
Citric acid	5.43 ± 0.03 ^c^	6.41 ± 0.00 ^a^	0.09 ± 0.00 ^d^	5.47 ± 0.01 ^b^	0.08 ± 0.00 ^d^
Lactic acid	0.26 ± 0.01 ^c^	0.40 ± 0.01 ^c^	4.26 ± 0.21 ^a^	0.41 ± 0.01 ^c^	2.89 ± 0.18 ^b^
Malic acid	0.51 ± 0.00 ^c^	0.70 ± 0.01 ^a^	0.18 ± 0.01 ^d^	0.55 ± 0.01 ^b^	0.19 ± 0.01 ^d^
Ascorbic acid	0.51 ± 0.01 ^b^	0.58 ± 0.01 ^a^	0.26 ± 0.00 ^d^	0.44 ± 0.01 ^c^	0.14 ± 0.00 ^e^

Different letters represented a significant difference within the same row (*p* < 0.05). Fresh: fresh YLL juice; TP: thermal processing treated YLL juice; F-TP: fermented thermal processing treated YLL juice; HHP: high hydrostatic pressure treated YLL juice; F-HHP: fermented high hydrostatic pressure treated YLL juice.

## Supplementary Materials

The following are available online at https://www.mdpi.com/2304-8158/8/8/308/s1. Figure S1: Growth changes of LAB in the fermentation of TP and HHP-treated YLL juice, Table S1: Individual FAA content in different treated YLL juice, Table S2: Volatile compounds identified by GC-MS analysis in the fresh, TP-treated, fermented TP-treated, HHP-treated and fermented HHP-treated YLL juice, Table S3: Cumulative contribution and predication performance (PCA, PLS-DA) of different treated YLL juice, Table S4: Potential markers of volatile components of different treatment of YLL juice.

## References

[1] Ankolekar C., Pinto M., Greene D., Shetty K. (2012). In vitro bioassay based screening of antihyperglycemia and antihypertensive activities of Lactobacillus acidophilus fermented pear juice. Innov. Food Sci. Emerg. Technol..

[2] Granato D., Branco G.F., Nazzaro F., Cruz A.G., Faria J.A. (2010). Functional Foods and Nondairy Probiotic Food Development: Trends, Concepts, and Products. Compr. Rev. Food Sci. Food Saf..

[3] Genta S., Cabrera W., Habib N., Pons J., Carillo I.M., Grau A., Sanchez S. (2009). Yacon syrup: Beneficial effects on obesity and insulin resistance in humans. Clin. Nutr..

[4] Ojansivu I., Ferreira C.L., Salminen S. (2011). Yacon, a new source of prebiotic oligosaccharides with a history of safe use. Trends Food Sci. Technol..

[5] Angelov A., Gotcheva V., Kuncheva R., Hristozova T. (2006). Development of a new oat-based probiotic drink. Int. J. Food Microbiol..

[6] Mikkelsen D., Flanagan B.M., Dykes G.A., Gidley M.J. (2010). Influence of different carbon sources on bacterial cellulose production by Gluconacetobacter xylinus strain ATCC 53524. J. Appl. Microbiol..

[7] Jagannath A., Raju P., Bawa A. (2010). Comparative evaluation of bacterial cellulose (nata) as a cryoprotectant and carrier support during the freeze drying process of probiotic lactic acid bacteria. LWT.

[8] Rodriguez H., Curiel J.A., Landete J.M., Rivas B.D.L., De Felipe F.L., Gómez-Cordovés C., Mancheño J.M., Muñoz R. (2009). Food phenolics and lactic acid bacteria. Int. J. Food Microbiol..

[9] Prado F.C., Parada J.L., Pandey A., Soccol C.R. (2008). Trends in non-dairy probiotic beverages. Food Res. Int..

[10] Barba F.J., Esteve M.J., Frígola A. (2012). High Pressure Treatment Effect on Physicochemical and Nutritional Properties of Fluid Foods during Storage: A Review. Compr. Rev. Food Sci. Food Saf..

[11] Li X., Farid M. (2016). A review on recent development in non-conventional food sterilization technologies. J. Food Eng..

[12] Yang Y., Xia Y., Wang G., Yu J., Ai L. (2017). Effect of mixed yeast starter on volatile flavor compounds in Chinese rice wine during different brewing stages. LWT.

[13] GB 4789.2-2016 National Food Safety Standard (2016). China: Food Microbiological Examination: Aerobic Plate Count. http://tradechina.dairyaustralia.com.au/wp-content/uploads/2018/08/GB-4789.2-2016-Safety-Standard-Food-Microbiological-Examination-Aerobic-Plate-Count-.pdf.

[14] GB 4789.35-2016 National Food Safety Standard (2016). China: Food Microbiological Examination: Lactic Acid Bacteria Plate Count. http://www.svscr.cz/wp-content/files/zivocisne-produkty/GB4789._35_2010_food_microbiological_examination_lactic_acid_bacteria.pdf.

[15] Yu Y., Xiao G., Xu Y., Wu J., Fu M., Wen J. (2015). Slight Fermentation with *Lactobacillus fermentium* Improves the Taste (Sugar:Acid Ratio) of Citrus (*Citrus reticulata* cv. chachiensis) Juice. J. Food Sci..

[16] Wu Y., Cui S.W., Tang J., Gu X. (2007). Optimization of extraction process of crude polysaccharides from boat-fruited sterculia seeds by response surface methodology. Food Chem..

[17] Ibegbulem C., Igwe C., Okwu G., Ujowundu C., Onyeike E., Ayalogu E. (2013). Total amino acid profiles of heat-processed fresh Elaeis guineensis and Raphia hookeri wines. Food Chem..

[18] Kaplan H., Hutkins R.W. (2000). Fermentation of Fructooligosaccharides by Lactic Acid Bacteria and Bifidobacteria. Appl. Environ. Microbiol..

[19] Reuss R., Stratton J., Smith D., Read P., Cuppett S., Parkhurst A. (2010). Malolactic Fermentation as a Technique for the Deacidification of Hard Apple Cider. J. Food Sci..

[20] Marsellés A.R. (2007). Effects of thermal and non-thermal processing treatments on fatty acids and free amino acids of grape juice. Food Control..

[21] Blandino A., Al-Aseeri M., Pandiella S., Cantero D., Webb C. (2003). Cereal-based fermented foods and beverages. Food Res. Int..

[22] Komata Y. (1969). The taste and constituents of foods. Nippon Shokuhin Kogyo Gakkaishi.

[23] Viljanen K., Lille M., Heiniö R.-L., Buchert J. (2011). Effect of high-pressure processing on volatile composition and odour of cherry tomato purée. Food Chem..

[24] Deng Y., Zhong Y., Yu W., Yue J., Liu Z., Zheng Y., Zhao Y. (2013). Effect of hydrostatic high pressure pretreatment on flavor volatile profile of cooked rice. J. Cereal Sci..

[25] Di Cagno R., Filannino P., Gobbetti M. (2017). Lactic acid fermentation drives the optimal volatile flavor-aroma profile of pomegranate juice. Int. J. Food Microbiol..

[26] Tripathi J., Chatterjee S., Gamre S., Chattopadhyay S., Variyar P.S., Sharma A. (2014). Analysis of free and bound aroma compounds of pomegranate (*Punica granatum* L.). LWT.

[27] Perez-Cacho P.R., Rouseff R. (2008). Processing and Storage Effects on Orange Juice Aroma: A Review. J. Agric. Food Chem..

[28] Jyoti B., Suresh A., Venkatesh K. (2004). Effect of preculturing conditions on growth of Lactobacillus rhamnosus on medium containing glucose and citrate. Microbiol. Res..

[29] Kaack K., Christensen L.P., Hughes M., Eder R. (2005). The relationship between sensory quality and volatile compounds in raw juice processed from elderberries (*Sambucus nigra* L.). Eur. Food Res. Technol..

[30] Bryant R.J., McClung A.M. (2010). Volatile profiles of aromatic and non-aromatic rice cultivars using SPME/GC-MS. Food Chem..

[31] Di Cagno R., Surico R.F., Paradiso A., De Angelis M., Salmon J.-C., Buchin S., De Gara L., Gobbetti M. (2009). Effect of autochthonous lactic acid bacteria starters on health-promoting and sensory properties of tomato juices. Int. J. Food Microbiol..

[32] Servili M. (2000). Relationships between the volatile compounds evaluated by solid phase microextraction and the thermal treatment of tomato juice: Optimization of the blanching parameters. Food Chem..

[33] Chen C., Zhao S., Hao G., Yu H., Tian H., Zhao G. (2017). Role of lactic acid bacteria on the yogurt flavour: A review. Int. J. Food Prop..

[34] Borade S.N., Deshmukh R.R. (2014). Comparative Study of Principal Component Analysis and Independent Component Analysis. Int. J. Comput Appl..

[35] Park S.-E., Yoo S.-A., Seo S.-H., Lee K.-I., Na C.-S., Son H.-S. (2016). GC–MS based metabolomics approach of Kimchi for the understanding of Lactobacillus plantarum fermentation characteristics. LWT.

