# Supplementary material for: High Hydrostatic Pressure and Co-Fermentation by Lactobacillus rhamnosus and Gluconacetobacter xylinus Improve Flavor of Yacon-Litchi-Longan Juice
Source: Foods. 2019 Aug 1;8(8):308. doi: 10.3390/foods8080308 (PMC6722649; doi:10.3390/foods8080308)
Supplement: Supplementary file 1 [file foods-08-00308-s001.pdf]

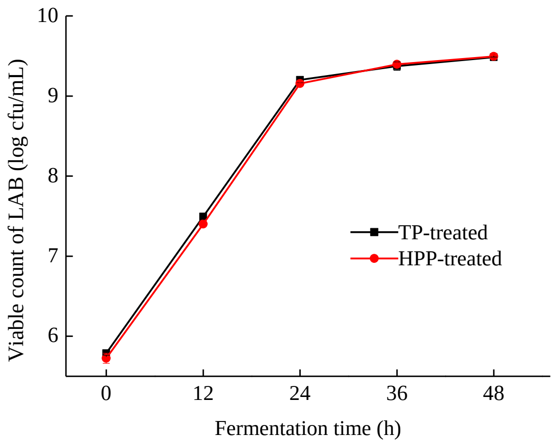

Figure S1. Growth changes of LAB in the fermentation of TP and HHP treated YLL juice.

Table S1. Individual FAA content in different treated YLL juice.

FAA content (mg/L)	Fresh	TP	F-TP	HHP	F-HHP
Phosphoserin (P-Ser)	0.00±0.00[c]	0.00±0.00[c]	61.05±0.41[b]	0.00±0.00[c]	75.21±2.27[a]
Taurine (Tau)	0.00±0.00[c]	0.00±0.00[c]	18.79±0.05[b]	0.00±0.00[c]	37.09±14.22[a]
Phosphorylethanolamine (PEA)	0.00±0.00[b]	0.00±0.00[b]	26.33±2.84[a]	0.00±0.00[b]	0.00±0.00[b]
Aspartic acid (Asp)	191.15±5.71[b]	251.24±36.42[a]	72.10±1.83[c]	192.63±19.40[b]	60.16±1.96[c]
Threonine (Thr)	29.36±1.02[b]	38.18±4.95[a]	3.44±0.14[c]	27.45±4.55[b]	6.40±1.09[c]
Serine (Ser)	137.36±3.64[b]	178.71±21.66[a]	29.28±0.59[c]	139.50±13.47[b]	35.56±0.07[c]
Glutamic acid (Glu)	315.56±10.67[c]	405.27±52.96[b]	474.11±12.48[ab]	268.17±33.98[c]	491.04±17.59[a]
α-Aminoadipic acid (a-AAA)	0.00±0.00[c]	0.00±0.00[c]	57.39±0.51[a]	0.00±0.00[c]	55.04±0.55[b]
Glycine (Gly)	36.03±0.74[b]	53.09±5.78[a]	24.85±2.48[c]	30.71±5.49[bc]	24.89±1.86[c]
Alanine (Ala)	874.89±20.15[b]	1144.41±132.72[a]	744.27±58.54[b]	892.71±83.55[b]	794.87±56.29[b]
α-Aminobutyric acid (a-ABA)	22.38±0.30[ab]	28.27±6.17[a]	23.04±2.54[ab]	18.48±2.47[b]	27.76±1.22[a]
Valine (Val)	112.72±2.94[a]	125.92±28.44[a]	55.49±3.93[b]	112.51±10.80[a]	56.42±4.52[b]
Methionine (Met)	25.33±0.69[b]	30.65±3.32[a]	4.74±0.58[c]	24.85±2.30[b]	5.24±0.61[c]
Isoleucine (Ile)	56.14±2.11[b]	70.20±9.56[a]	9.73±0.19[c]	56.10±5.57[b]	0.00±0.00[c]
Leucine (Leu)	101.98±3.65[b]	131.22±17.41[a]	33.66±0.23[c]	102.08±10.40[b]	28.93±0.73[c]
Tyrosine (Tyr)	36.71±1.90[a]	41.92±9.50[a]	0.00±0.00[b]	36.29±4.43[a]	0.00±0.00[b]
Phenylalanine (Phe)	29.07±0.94[b]	36.83±4.86[a]	3.78±0.20[c]	28.62±3.22[b]	6.75±0.61[c]
b-Alanine (b-Ala)	7.46±2.42[b]	11.36±1.78[b]	27.54±3.25[a]	9.18±0.91[b]	29.60±2.14[a]
g-Aminobutyric acid (g-ABA)	387.36±9.13[b]	493.48±56.94[a]	347.15±23.21[b]	385.64±34.27[b]	366.91±21.85[b]
Ethanolamine (EOHNH2)	11.7±0.29[ab]	15.31±1.50[a]	10.45±1.24[b]	12.28±0.85[ab]	13.45±0.33[ab]
Ornithine (Orn)	0.00±0.00[b]	0.00±0.00[b]	229.22±12.74[a]	0.00±0.00[b]	238.14±15.27[a]
Lysine (Lys)	25.96±1.03[a]	29.43±7.47[a]	0.00±0.00[b]	25.81±3.01[a]	0.00±0.00[b]
Histidine (His)	0.00±0.00[b]	13.87±0.73[a]	0.00±0.00[b]	0.00±0.00[b]	0.00±0.00[b]
Arginine (Arg)	424.89±17.04[b]	539.92±72.98[a]	0.00±0.00[c]	420.22±50.40[b]	0.00±0.00[c]
Proline (Pro)	111.75±1.72[b]	139.74±15.87[a]	100.24±4.07[b]	110.96±9.52[b]	104.84±0.32[b]
Total amino acids	2894.62±21.02[b]	3777.22±15.56[a]	2353.07±4.46[d]	2892.39±11.24[b]	2494.58±12.35[c]

[a, b, c, d] Different letters represented a significant difference within the same row ($p < 0.05$).

Table S2. Volatile compounds identified by GC-MS analysis in the fresh, TP-treated, fermented TP-treated, HHP-treated and fermented HHP-treated YLL juice.

Number	Volatile coumpounds	Retention index	Fresh	TP	F-TP	HHP	F-HHP
	Alcohol						
1	Ethyl alcohol	445	X[1)]	X	X	X	X
2	3-Methyl-1-butanol	734	X	–[2)]	–	X	–
3	2-Methylbutan-1-ol	–	X	–	–	X	–
4	(R,R)-2,3-Butanediol	807	–	–	X	–	X
5	trans-2-Hexen-1-ol	865	X	X	–	X	X
6	Heptanol	971	X	–	–	X	–
7	6-methyl-5-hepten-2-ol	994	X	–	X	–	–
8	6-Hepten-1-ol, 2-methyl-	994	–	–	–	X	X
9	5-ethenyltetrahydro-.alpha.,.alpha.-5-trimethyl-, cis-2-Furanmethanol	1071	X	X	–	X	–
10	1-Octanol	1072	–	–	–	X	–
11	Linalool	1102	X	X	X	X	X
12	Phenethyl alcohol	1114	X	X	X	X	X
13	Bicyclo[3.1.1]hept-3-en-2-ol,4,6,6-trimethyl-	1146	–	X	–	X	–
14	Neroloxide	1152	X	–	X	–	X
15	Borneol	1173	–	–	X	–	–
16	Menthol	1179	–	X	–	–	–
17	Terpinen-4-ol	1181	X	X	X	X	X
18	2-(4-Methylphenyl)propan-2-ol	1189	–	X	X	X	X
19	α-Terpineol	1196	X	–	–	X	X
20	2,6-Dimethyl-3,5,7-octatriene-2-ol, ,E,E-	1210	X	–	–	X	–
21	2,6-Octadien-1-ol,3,7-dimethyl-, (2Z)-	1226	X	–	X	–	X
22	6-Octen-1-ol,3,7-dimethyl-, (3R)-	1228	X	–	X	X	X
23	iso-Geraniol	1241	X	–	X	–	X
24	Geraniol	1252	X	X	X	X	X
	Ester						
25	Ethyl Acetate	625	X	X	–	X	X
26	2-Butenoic acid, ethyl ester	842	X	–	–	X	–

27	Ethyl 2-methylbutyrate	846	X	–	–	X	–
28	Methyl hexanoate	924	X	–	–	–	–
29	Ethyl 3-hydroxybutyrate	936	X	X	X	X	X
30	Ethyl caproate	1000	–	–	X	–	X
31	Methyl benzoate	1095	X	–	–	X	X
32	Ethyl benzoate	1171	X	–	–	–	–
33	Methyl salicylate	1192	X	X	X	X	X
34	Methyl 2-methoxybenzoate	1336	X	–	–	X	X
35	Carbamodithioic acid,N,N-diethyl-, methyl ester	1376	X	X	X	X	X
36	Ethyl caprate	1396	X	–	–	–	–
37	Ethyl laurate	1595	X	X	–	–	–
	Aldehyde						
38	Isovaleraldehyde		–	X	–	–	–
39	Hexanal	803	X	X	–	X	–
40	trans-2-Hexenal	851	X	–	–	X	–
41	2-Hexenal	854	–	X	–	–	–
42	Heptaldehyde	903	–	–	–	X	–
43	2-Heptenal, (2Z)-	958	X	–	–	X	–
44	Benzaldehyde	966	X	X	X	X	X
45	Octanal	1005	X	X	–	X	–
46	(E)-2-Octenal	1059	–	–	–	X	–
47	Nonanal	1106	X	X	–	X	–
48	Ampholenic aldehyde	1128	–	–	–	X	–
49	3-Ethylbenzaldehyde	1165	X	X	–	X	–
50	2,4-Dimethylbenzaldehyde	1219	X	–	–	–	–
51	Citral	1270	–	–	–	–	X
52	3-Ethylbenzaldehyde	1277	–	X	–	–	–
	Ketone						
53	2,3-Butanedione	–	–	–	X	–	X
54	3-Hydroxy-2-butanone	–	–	–	X	–	X
55	2-Heptanone	890	–	–	X	–	X
56	6-Methyl-5-hepten-2-one	985	X	X	X	X	X
57	2,5-Hexanedione, 3,4-dihydroxy-3,4-dimethyl-	1063	–	–	X	–	X
58	2-Nonanone	1092	–	–	X	–	X

59	(R,S)-5-Ethyl-6-methyl-3E-hepten-2-one	1141	–	–	–	X	–
60	Bicyclo[3.1.1]heptan-3-one,6,6-dimethyl-2-methylene-	1163	X	–	–	X	–
61	4,6,6-trimethyl-bicyclo[3.1.1]hept-3-en-2-one	1208	X	–	–	X	X
62	2-Undecanone	1295	–	–	X	–	–
63	Geranylacetone	1449	–	–	–	–	X
64	4-[2,2,6-trimethyl-7-oxabicyclo[4.1.0]hept-1-yl]-3-Buten-2-one	1481	–	–	–	X	–
	Acid						
65	Acetic acid	688	X	X	X	–	X
	Terpene hydrocarbons						
66	Trichloromethane	–	X	X	–	X	–
67	3-Methyl-hexane	–	–	X	–	X	–
68	Cyclopentane,1,2-dimethyl-	–	–	X	–	–	–
69	Heptane	–	–	X	–	X	X
70	Methylcyclohexane	–	–	X	–	X	–
71	Toluene	–	–	X	–	–	–
72	Ethylbenzene	858	–	X	X	X	X
73	p-Xylene	869	–	X	X	–	X
74	phenylethylene	892	–	X	X	X	X
75	(1S)-(-)-alpha-Pinene	932	X	X	–	X	–
76	(1S)-(1)-beta-Pinene	976	X	–	–	–	–
77	Decane	1001	–	X	–	X	–
78	1,2,3-trimethyl-Benzene	969	X	–	–	–	–
79	1-methyl-3-(1-methylethyl)-benzen	1025	–	–	–	X	X
80	Cyclohexene,1-methyl-4-(1-methylethenyl)-, (4R)-	1029	X	X	X	–	–
81	(Z)-13,7-dimethyl-3,6-octatriene	1047	X	X	–	X	–
82	1-methyl-4-(1-methylethenyl)-Benzene	1092	X	–	–	X	–
83	(3E,5E)-2,6-Dimethyl-1,3,5,7-octatetrene	1130	X	–	–	X	–
84	3,6-Dimethyl-	1155	–	X	X	X	X

	2,3,3a,4,5,7a-hexahydrobenzofuran						
85	Benzene,1,3-bis(1,1-dimethylethyl)-	1247	–	X	X	X	X
86	Bicyclo[7.2.0]undec-4-ene,4,11,11-trimethyl-8-methylene-,(1R,4Z,9S)-	1418	X	–	–	–	–
87	Naphthalene,1,2,3,5,6,7,8,8a-octahydro-1-methyl-6-methylene-4-(1-methylethyl)-	1429	X	X	X	–	–
88	Naphthalene,1,2,4a,5,6,8a-hexahydro-4,7-dimethyl-1-(1-methylethyl)-	1474	X	X	X	–	X
89	1,6-Cyclodecadiene,1-methyl-5-methylene-8-(1-methylethyl)-,(1E,6E,8S)-	1480	X	–	–	–	–
90	α-muurolene	1498	X	X	X	X	X
91	d-Cadinene	1517	X	X	X	–	–
	Others						
92	Oxazole,2,4,5-trimethyl-	845	–	–	X	–	X
93	2H-Pyran,tetrahydro-4-methyl-2-(2-methyl-1-propen-1-yl)-	1111	X	–	–	–	–
94	Epizonarene	1368	X	–	X	X	X
95	2,4-Di-tert-butylphenol	1506	–	X	X	X	X

[1]“X” means the compounds were detected.

[2]“–” indicates the compounds were not detected.

Table S3. Cumulative contribution and predication performance (PCA, PLS-DA) of different treated YLL juice.

	**Component**	**1**	**2**	**3**	**4**
PCA	R^2X	0.417	0.673	0.884	0.966
	Q^2	0.231	0.160	0.626	0.859
PLS-DA	R^2X	0.245	0.500	0.749	0.997
	Q^2	0.050	0.112	0.303	0.987

Table S4. Potential markers of volatile components of different treatment of YLL juice.

	number	volatile compounds	VIP value	Aroma character
Fresh	Q3	trans-2-Hexenal	1.06804	Green leaf flavor
	C5	trans-2-Hexen-1-ol	1.30718	Immature fruit odor
	C11	Linalool	2.02993	Floral aroma
	C19	α-Terpineol	1.1555	Lilac flavor
	X10	(1S)-(-)-alpha-Pinene	1.8553	Resin fragrance with cool pine needle odor
	X16	(Z)-13,7-dimethyl-3,6-octatriene	2.00629	Grassy aroma
TP	X3	Cyclopentane,1,2-dimethyl-	1.18452	fatty liked odor
	X4	Heptane	1.45127	soapy-liked odor
	X5	Methylcyclohexane	1.47531	fatty and soapy-liked odor
F-TP	T1	2,3-Butanedione	2.60679	Fruity aroma
	T2	3-Hydroxy-2-butanone	3.23883	Milk aroma
	S	Acetic acid	1.16973	Harsh odor
HHP	Q2	Hexanal	3.13027	Grassy aroma
	Q5	Heptaldehyde	1.39762	Fruit flavor
	C10	1-Octanol	1.04963	Lemon odor
F-HHP	C8	6-Hepten-1-ol, 2-methyl-	1.21224	
	C17	Terpinen-4-ol	1.04081	Warm pepper, slightly earthy
	C24	Geraniol	1.37712	Floral fragrance
	Z1	Ethyl Acetate	1.9947	A fruity wine aroma
	Z6	Ethyl caproate	2.19416	Fruit aroma
	X8	p-Xylene	1.33859	
	X9	Phenylethylene	1.58358	Light sweetness aroma
